# Supplementary material for: Long-term participation in collaborative fisheries research improves angler opinions on marine protected areas
Source: PeerJ. 2020 Oct 28;8:e10146. doi: 10.7717/peerj.10146 (PMC7602691; doi:10.7717/peerj.10146)
Supplement: Supplemental Information 2 [file peerj-08-10146-s002.pdf]

Survey Questionnaire (minus screening question, minus letter of consent)

CCFRP Volunteer Angler Survey

This survey consists of a series of questions arranged in four sections:

I. CCFRP Volunteering
II. Fisheries Management and Health of CA Groundfish Stocks
III. (A,B, and **C)** Marine Protected Areas (MPAs)
IV. Demographics and Miscellaneous Questions

Since CCFRP was expanded statewide in 2017, for the purpose of this survey please respond about your involvement between 2007 and 2016. Your answers will be kept anonymous.

Please complete this survey in one sitting. Your response will only be recorded if you click "Submit" at the end of the survey. This survey should take 15 minutes to complete.

Section I. CCFRP Volunteering

The following questions ask about your experiences as a volunteer angler with CCFRP *between 2007 and 2016*:

1) What year did you start volunteering with CCFRP?

____

2) How many years did you volunteer with CCFRP?

____

3) On average, how many CCFRP trip(s) did you go on per year? *(give your best guess)*

____

4) Why did you choose to become a volunteer angler? *(select all that apply)*
- ○ To give back to fisheries resources
- ○ To participate in science
- ○ To fish inside marine protected areas (MPAs)
- ○ To spend time with friends/family
- ○ To enjoy a day of fishing provided by CCFRP
- ○ Other:_______________

5) Do you plan to continue volunteering with CCFRP?
- ○ Yes
- ○ No

[IF "YES," continue QUESTION 5A.1, IF "NO," skip to QUESTION 5A.2]

5A.1) Why do you continue volunteering with CCFRP? *(select all that apply)*

- ○ To give back to fisheries resources
- ○ To participate in science
- ○ To fish inside marine protected areas (MPAs)
- ○ To spend time with friends/family
- ○ To enjoy a day of fishing provided by CCFRP
- ○ Other:_______________

[CONTINUE TO Question 6]

5A.2) Why did you stop volunteering with CCFRP? *(select all that apply)*

- ○ I moved away
- ○ I no longer had the time
- ○ The trips I could go on were already full
- ○ Personal reasons (health, family, etc.)
- ○ I had issues with other volunteers and/or staff
- ○ I had issues with the science being conducted (protocol, sampling, etc.)
- ○ I did not realize I could continue volunteering
- ○ Other:_____________ *[CONTINUE TO Question 6]*

6) Have you attended a CCFRP "Volunteer Appreciation and Data Workshop" event?

- ○ Yes
- ○ No

[IF "YES," continue to QUESTION 6A) IF "NO," skip to SECTION II, QUESTION 7]

6A) How many Volunteer Appreciation and Data Workshop events have you attended?

____

6B) Why did you attend the Volunteer Appreciation and Data Workshop event(s)? *(select all that apply)*

- ○ To learn if there are effects of MPA creation on fish populations
- ○ To see data from the program
- ○ To learn about fisheries resources
- ○ To participate in community events
- ○ To see friends/family
- ○ To talk to CCFRP staff
- ○ To enjoy food and/or raffle prizes provided by CCFRP
- ○ To see videos/pictures of trips
- ○ Other:____________

[CONTINUE TO SECTION II]

Section II. Fisheries Management and Health of CA Groundfish Stocks

The following questions ask about any experience you may have had in fisheries management prior to volunteering with CCFRP, as well as your current opinions of the health and management of groundfish in California.

For this survey, "groundfish" is defined as all species of rockfish, lingcod, cabezon, and flatfish.

7) In your opinion, what is the current overall health of California groundfish stocks?
I believe California groundfish stocks to be…

- ○ Very healthy
- ○ Somewhat healthy
- ○ Neutral
- ○ Somewhat unhealthy
- ○ Very unhealthy
- ○ I don't know

8) In your opinion, how well are California groundfish stocks managed?
I believe California groundfish stocks are...

- ○ Very well managed
- ○ Well managed
- ○ Adequately managed
- ○ Poorly managed
- ○ Very poorly managed
- ○ I don't believe they are managed at all
- ○ I don't know

9) In your opinion, are the following recreational fisheries management strategies effective, or ineffective, for ensuring healthy groundfish stocks?

Management strategy	Effective	Not Effective	Not sure
Minimum size limits	○	○	○
Season closures	○	○	○
Spatial closures (e.g. rockfish conservation areas, etc.)	○	○	○
Depth restrictions	○	○	○
Catch (bag) limits	○	○	○

10) Have you ever worked in marine resource management at the local, state, or federal level? *(for example, at CA Department of Fish and Wildlife or NOAA)*

- ○ Yes
- ○ No

11) Before volunteering with CCFRP, did you have an opinion of the quality of data used in fisheries management decisions?

- ○ Yes
- ○ No

[IF "YES," continue to QUESTION 11A, IF "NO," skip to SECTION III, QUESTION 12]

11A) Has volunteering with CCFRP changed your opinion of the quality of these data?

- ○ Yes
- ○ No

[IF "YES," continue to QUESTION 11B, IF "NO," skip to SECTION III, QUESTION 12]

11B) In what way did volunteering with CCFRP change your opinion of the quality of these data?

My opinion changed to be...

- ○ More positive
- ○ Positive from negative
- ○ Negative from positive
- ○ More negative

[CONTINUE to SECTION III.]

Section III.A. Marine Protected Areas (MPAs)

*The Marine Life Protection Act (MLPA) was passed in 1999, requiring "the state to redesign its previously existing system of...marine protected areas (MPAs)...to increase its coherence and effectiveness at protecting the state's marine life, habitats, and ecosystems."**

**California Department of Fish and Wildlife. 2016. "2016 Master Plan for Marine Protected Areas- Appendix E."*

The following questions ask about your experiences with marine protected areas (MPAs) in California.

12) Did you participate in the Marine Life Protection Act (MLPA) planning process at any time between 1999 and 2012? *(for example, by attending a meeting, being part of the Regional Stakeholder Group, submitting a public comment, etc.)*

- ○ Yes
- ○ No

13) Did you ever fish in areas that are now MPAs?

- ○ Yes
- ○ No
- ○ I don't know

[IF "YES," continue to QUESTION 13A, IF "NO," skip to QUESTION 14]

13A) In which areas that are now Central Coast MPAs did you previously fish?

- ○ Año Nuevo State Marine Reserve
- ○ Point Lobos State Marine Reserve
- ○ Piedras Blancas State Marine Reserve
- ○ Point Buchon State Marine Reserve
- ○ Other(s):____
- ○ I don't know

____

____

Section III.B. Marine Protected Areas (MPAs) continued

The following questions ask about your opinions of marine protected areas (MPAs) in California *BEFORE* volunteering with CCFRP.

For this survey, "groundfish" is defined as all species of rockfish, lingcod, cabezon, and flatfish.

14) Before volunteering with CCFRP, what was your general opinion of the creation of MPAs in California?
My general opinion of MPA creation was...

- ○ Positive
- ○ Somewhat positive
- ○ Somewhat negative
- ○ Negative
- ○ No opinion

15) Before volunteering with CCFRP, did you believe the creation of MPAs would affect the abundance of groundfish inside MPAs?

- ○ Yes
- ○ No
- ○ I don't know

[IF "YES," continue to QUESTION 15A, IF "NO," skip to QUESTION 16]

15A) What effect did you believe MPA creation would have on groundfish abundance?

I believed there would be a...

- Large increase
- Small increase
- Small decrease
- Large decrease

16) Before volunteering with CCFRP, did you believe the creation of MPAs would affect the size of groundfish caught inside MPAs?

- Yes
- No
- I don't know

[IF "YES," continue to QUESTION 16A, IF "NO," skip to SECTION IIIC, QUESTION 17]

16A) What effect did you believe MPA creation would have on groundfish size?
I believed there would be a…

- Large increase
- Small increase
- Small decrease
- Large decrease

Section III.C. Marine Protected Areas (MPAs) continued

The following questions ask about your opinions of marine protected areas (MPAs) in California *AFTER* volunteering with CCFRP.

For this survey, "groundfish" is defined as all species of rockfish, lingcod, cabezon, and flatfish.

17) Which type(s) of sampling sites did you visit with CCFRP trips? *("Reference" sites are CCFRP sampling sites with habitats similar to corresponding MPA sampling sites.)*

- MPA
- Reference
- Both
- I don't know

[IF "MPA, or Both" continue to QUESTION 17A, IF "Reference" or "I don't know" skip to QUESTION 18]

17A) Which Central Coast MPA site(s) did you visit with CCFRP between 2007 and 2016? *(select all that apply)*

- Año Nuevo State Marine Reserve
- Point Lobos State Marine Reserve
- Piedras Blancas State Marine Reserve
- Point Buchon State Marine Reserve
- I don't know

18) After volunteering with CCFRP, what is your general opinion of the creation of MPAs in California?
My general opinion of MPA creation is...
- ○ Positive
- ○ Somewhat positive
- ○ Somewhat negative
- ○ Negative
- ○ No opinion

19) After volunteering with CCFRP, do you believe the creation of MPAs affects the abundance of groundfish inside MPAs?
- ○ Yes
- ○ No
- ○ I don't know

[IF "YES," continue to QUESTION 19A, IF "NO," skip to QUESTION 20]

19A) What effect do you believe MPA creation has on groundfish abundance?
I believe there is a...
- ○ Large increase
- ○ Small increase
- ○ Small decrease
- ○ Large decrease

20) After volunteering with CCFRP, do you believe the creation of MPAs affects the size of groundfish caught inside MPAs?
- ○ Yes
- ○ No
- ○ I don't know

[IF "YES," continue to QUESTION 20A, IF "NO," skip to QUESTION 21]

20A) What effect do you believe MPA creation has on groundfish size?
I believe there is a...
- ○ Large increase
- ○ Small increase
- ○ Small decrease
- ○ Large decrease

21) If you believe that California MPA creation has affected groundfish abundance and/or size, were these effects faster or slower than you anticipated?
- ○ Faster
- ○ Slower
- ○ As expected
- ○ I don't know
- ○ N/A, I do not believe there is an effect

22) If you believe that California MPA creation affected groundfish abundance and/or size, what aspect(s) of MPAs do you believe caused these effect(s)? *(select all that apply)*

- ○ Location of MPAs
- ○ Size of MPAs
- ○ Enforcement of MPA restrictions
- ○ Planning of MPAs as a network
- ○ MPA protection of a portion of fish populations
- ○ Voluntary compliance with restrictions
- ○ Other:________
- ○ N/A, I do not believe there is an effect

[CONTINUE to SECTION IV.]

Section IV. Demographics and Miscellaneous Questions

The following demographic questions ask for some general information about you.

23) What is your age?

- ○ 18-24 years old
- ○ 25-34 years old
- ○ 35-44 years old
- ○ 45-54 years old
- ○ 55-64 years old
- ○ 65-74 years old
- ○ 75 years or older

24) What is your gender?

- ○ Female
- ○ Male
- ○ Other
- ○ I prefer not to say

25) At what age did you start salt-water angling?

____

26) On average how many recreational angling trips do you go on per year? (*do not include CCFRP trips)*

27) In general, would you say that you are more conservation minded or less conservation minded than others in the recreational angling community?
Compared to the rest of the recreational angling community, I am...

- ○ More conservation minded
- ○ Similarly conservation minded
- ○ Less conservation minded
- ○ I don't know

28) Have you ever worked in the *recreational* fishing industry? *(for example, as a captain, boat crew, bait or tackle salesperson, etc.)*

- ○ Yes
- ○ No

29) Have you ever worked in the *commercial* fishing industry? *(for example, as a fisherman, captain, boat crew, buyer, etc.)*

- ○ Yes
- ○ No

You have reached the end of the survey!
If you are finished, click "Submit" to record your answers.

[If done, click "Submit" to submit survey]

Your response has been submitted and your answers recorded.
Thank you for taking the time to fill out this survey and for all of your help as a volunteer with CCFRP!

[End of Survey]
